# The Knowledge and Preparedness of General Dentists about Medical Emergencies in Iran

**Published:** 2011-03-01

**Authors:** M Amirchaghmaghi, J Sarabadani, Z Delavarian, P Mosannen Mozafary, A Shahri, Z Dalirsani

**Affiliations:** 1 Department of Oral Medicine, Dental Research Center, School of Dentistry, Mashhad University of Medical Sciences, Mashhad, Iran; 2 Dentist, Mashhad University of Medical Sciences, Mashhad, Iran

**Keywords:** Knowledge, Preparedness, Medical emergencies, Dentist, Iran

Dear Editor,

About one out of three patients referred to dental clinics have a medical problem with most common being cardiac, respiratory, and cerebrovascular diseases.[1],[2]

As a health care provider, dentists must be prepared for prevention, diagnosis and treatment of medical emergencies (MEs) in dental office, by means of basic recommended emergency equipments, emergency drugs and an appropriate knowledge. To assess the knowledge and preparedness of Mashhad (in north east of Iran) general dentists about common MEs in dental settings, a cross sectional, analytical study was performed using a self-stablished questionnaire. The validity and reliability of the questionnaire was approved by Research Association of Mashhad Dental School. We randomly selected 186 dentists (p=0.5, α=0.05) using a randomization software (Available at:HTTP://WWW.Randomization.com). With the exception of name, other demographic data such as sex, age and graduating university were obtained. The Ethics Committee of Mashhad University of Medical Sciences approved the study protocol. The first section of questionnaire consisted of 20 knowledgebased questions; with one score given for each correct answer. The maximum possible score for the knowledge component of the questionnaire was 20. The scores of knowledge were classified to excellent (17- 20), good (14-17), moderate (10-14) and poor (0-10).

**Fig. 1:**
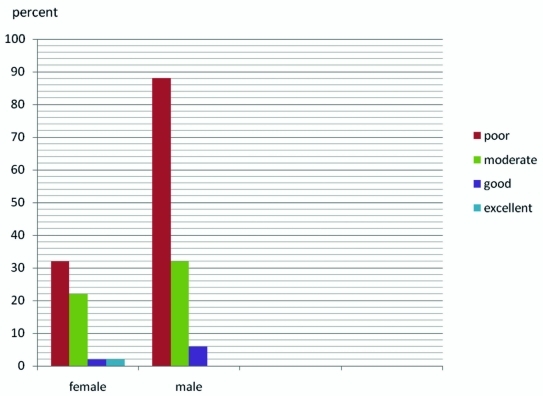
Different levelof knowledge about Medical Emergencies among general dentists.

The participants’ preparedness towards MEs was assessed with 20 questions with 0.5 to 2 degree given for correct answer based on importance of questions. The total score was 30 and scores of preparedness were classified to excellent (25-30), good (20-24), moderate (15-19) and poor (0-14). The collected results were analysed using SPSS software (version 15, Chicago, IL, USA). Descriptive statistics and analysis of variance were carried out, and a value of p< 0.05 was considered significant. Among 186 general dentists, 31.7% were female and 68.3% were male .The mean age was 40.92±8.22 years. (F: 38.48±7.55 and M: 42.05±8.30 years). Most of dentists were graduated from Mashhad Dental School (77.7%) and the remained from other universities. Regarding medical emergencies, the dentists’ knowledge was poor. Only 1.1% of them had excellent knowledge (17-20 degrees). Of the reminder, 4.3% had good, 29.3% had moderate and 65.2% had poor knowledge. The female dentists’ knowledge was higher than males (p=0.019) (Figure 1). There was a negative correlation between age and knowledge (p=0.001) and with an increase in age, the knowledge decreased. Most of dentists (66.7%) had a poor preparedness about MEs .Only in 0.5% of dentists, their preparedness was excellent, 4.3% had good and 28.5% had moderate preparedness. Males had significantly higher preparedness for management of medical emergencies than females (p=0.010). Seventy one percent of dentists received nitroglycerin in dental clinics. Other emergency drugs were defined in less quantities (aromatic ammoniac was seldom used). The most common equipment was syringe and needle for injection (63.4%) and ambubag was used only by 6.5% of dentists.

Our study denoted to a poor knowledge about MEs in Mashhad general dentists. The best knowledge was about diabetic emergencies and asthma and the worst knowledge was about adrenal insufficiency and foreign body aspiration. Birang et al. showed that the knowledge score of Esfahan dentists was 5.42/10.[3] Mollashahi et al. demonstrated that the knowledge of 96.8% of Zahedan dentists was from moderate to good.[4] In Behnia and Roshad study, the knowledge of 56.7% of Tehran dentists was from good to excellent.[5] So the knowledge of Mashhad dentists was lower than other provinces of Iran. This necessitates a rigorous revision in educational programs and a re-evaluation in dentistry course plans.

The preparedness was poor in 66.7% of our dentists. In other studies such as Timermann,[6] 50% of participants were able to manage medical emergencies. Gonzana study revealed that 59% of dentists judged themselves to be able to define CPR. Although only 46% had a correct concept; 54% were able to perform CPR.[7] These results highlight a need for higher educational plans on ME.

Fortunately, important emergency drugs such as oxygen, adrenaline and nitroglycerin are available in most of emergency kits of Mashhad dental clinics based on self reports of dentists. These results are similar to Mesgarzadeh study in Tabriz (Northwest of Iran).[8] It should be emphasized that lack of knowledge on EM, has mde these drug useless. So educational programs and materials such as posters and pamphlets on MEs can be of great benefit.
